# Loop-Mediated Isothermal Amplification for Rickettsia typhi (the Causal Agent of Murine Typhus): Problems with Diagnosis at the Limit of Detection

**DOI:** 10.1128/JCM.02786-13

**Published:** 2014-03

**Authors:** Sabine Dittrich, Josée Castonguay-Vanier, Catrin E. Moore, Narongchai Thongyoo, Paul N. Newton, Daniel H. Paris

**Affiliations:** a Lao-Oxford-Mahosot Hospital–Wellcome Trust Research Unit (LOMWRU), Microbiology Laboratory, Mahosot Hospital, Vientiane, Lao People's Democratic Republic; b Centre for Tropical Medicine, Nuffield Department of Medicine, Churchill Hospital, University of Oxford, Oxford, England, United Kingdom; c Mahidol-Oxford Tropical Medicine Research Unit (MORU), Faculty of Tropical Medicine, Mahidol University, Bangkok, Thailand

## Abstract

Murine typhus is a flea-borne disease of worldwide distribution caused by Rickettsia typhi. Although treatment with tetracycline antibiotics is effective, treatment is often misguided or delayed due to diagnostic difficulties. As the gold standard immunofluorescence assay is imperfect, we aimed to develop and evaluate a loop-mediated isothermal amplification (LAMP) assay. LAMP assays have the potential to fulfill the WHO ASSURED criteria (affordable, sensitive, specific, user friendly, robust and rapid, equipment free, deliverable to those who need them) for diagnostic methodologies, as they can detect pathogen-derived nucleic acid with low technical expenditure. The LAMP assay was developed using samples of bacterial isolates (*n* = 41), buffy coat specimens from R. typhi PCR-positive Lao patients (*n* = 42), and diverse negative controls (*n* = 47). The method was then evaluated prospectively using consecutive patients with suspected scrub typhus or murine typhus (*n* = 266). The limit of detection was ∼40 DNA copies/LAMP reaction, with an analytical sensitivity of <10 DNA copies/reaction based on isolate dilutions. Despite these low cutoffs, the clinical sensitivity was disappointing, with 48% (95% confidence interval [95% CI], 32.5 to 62.7%) (specificity, 100% [95% CI, 100 to 100%]) in the developmental phase and 33% (95% CI, 9.2 to 56.8%) (specificity, 98.5% [95% CI, 97.0% to 100%]) in the prospective study. This low diagnostic accuracy was attributed to low patient R. typhi bacterial loads (median, 210 DNA copies/ml blood; interquartile range, 130 to 500). PCR-positive but LAMP-negative samples demonstrated significantly lower bacterial loads than LAMP-positive samples. Our findings highlight the diagnostic challenges for diseases with low pathogen burdens and emphasize the need to integrate pathogen biology with improved template production for assay development strategies.

## INTRODUCTION

Murine typhus, an acute febrile illness with worldwide distribution, is increasingly recognized as an important cause of fever (1–3). The agent, Rickettsia typhi, is transmitted to humans by flea bites and/or by self-inoculation, from scratching of flea feces on the human skin (4, 5). Murine typhus and scrub typhus, caused by Orientia tsutsugamushi, are important causes of fever in Southeast Asia and represent up to 28% of blood culture-negative febrile cases in Lao and adjoining countries (6, 7).

The laboratory diagnosis of murine typhus is conventionally based on the indirect immunofluorescent assay (IFA) using paired serum samples. However, IFA is hampered by subjectivity of result interpretation and a lack of evidence-based interpretation guidelines in areas of endemicity where substantial background antibody levels confuse the interpretation of results, making single readings unreliable (8). A 4-fold titer increase in paired samples is an accepted diagnostic positivity criterion but remains retrospective by nature and may result in delayed antibiotic treatment. A clinically useful diagnostic test should enable early, sensitive, and specific detection of R. typhi, preferably at the point of care (POC). Cell culture confirms the presence of R. typhi with high specificity but with low sensitivity. Direct antigen- or DNA-based detection methods offer new, more specific diagnostic targets than serology assays. Quantitative real-time PCR (qPCR) assays (9, 10) can improve early diagnosis of rickettsial infections and, when combined with serological tests, expand the time frame of adequate diagnostic coverage (11).

Loop-mediated isothermal amplification (LAMP) is a practical and inexpensive methodology in comparison to related PCR methods and does not require a thermocycler (12, 13). These advantages make LAMP a strong contender for fulfilling the World Health Organization ASSURED criteria (affordable, sensitive, specific, user friendly, robust and rapid, equipment free, deliverable to those who need them) (12). Recent technological developments have led to major improvements, such as advanced heating devices (14, 15), optimized and simplified extraction technologies (16–18), and innovative methods for endpoint interpretation (19–21).

We therefore aimed to develop a LAMP assay and examine its diagnostic accuracy in relation to bacteria load for the early detection of R. typhi. If LAMP is of sufficient diagnostic accuracy, it may serve as a POC assay in central and peripheral health facilities to aid timely identification of murine typhus patients.

## MATERIALS AND METHODS

### LAMP primer design.

Five Rickettsia species genomes were downloaded from GenBank and aligned using the Artemis Comparison Tool (Sanger Institute, http://webact.org/WebACT/home) (22); they included R. felis strain URRWXCal2 (GenBank accession number CP000053), R. conorii strain Malish 7 (GenBank accession number AE006914), R. bellii strain RML369-C (GenBank accession number CP000087), R. prowazekii strain Madrid E (GenBank accession number AJ235269), and R. typhi strain Wilmington (GenBank accession number AE017197.1). A unique genetic region specific to R. typhi was identified in the cell surface-associated protein 1 gene (*sca1*, bp 14700 to 15000), and the *sca1* gene sequences available in GenBank in January 2011 were downloaded, aligned, and cropped (CLC Sequence Viewer, version 6.4 [CLC bio], and MUSCLE [multiple sequence comparison by log expectation] alignment tool). A highly specific set of LAMP primers, including loop primers (Table 1), was designed within a region 232 bp in length between F3 and B3 using Primer Explorer software on the Eiken Homepage (http://primerexplorer.jp/e/).

**TABLE 1:** Overview of the nucleotide sequences of the *sca1* LAMP primers developed in this study

Primer	Sequence (5′–3′)
F3	AGTAGGAGCGGTAATGGC
B3	GCACAACGATTCGGTAGTC
FIP	ACGCTTGATTGTGAAAATTTGAGCTGTTGAAGGAATTGCTATGG
BIP	ATCAGTACAACACAGGAAACTAACAGCTACCTCTTCTGTCATGTC
LF	TCGGTACAAAATGCCTTTTTATCT
LB	ACTTATCTAACAATGTGCAAAGCA

### Clinical specimens.

Samples were from patients recruited, as a part of fever studies, at Mahosot Hospital, Vientiane, Lao (a primary-tertiary hospital in the capital city of Lao with ∼400 beds) (23), and Salavan Provincial Hospital (with 70 beds, southern Lao) (1). Ethical approval for these investigations was granted by the Lao National Ethics Committee for Health Research and OXTREC, United Kingdom, and patients gave written informed consent before recruitment.

### Development phase.

Patients with R. typhi qPCR-positive (qPCR+) buffy coat samples (*n* = 42), healthy blood bank controls (*n* = 12), and febrile controls (*n* = 35) were included in the development phase (*n* = 89). The febrile control patients were all R. typhi qPCR negative (qPCR−) and included qPCR-confirmed patients with scrub typhus (*n* = 16) (10), dengue (*n* = 4) (24), leptospirosis (*n* = 3) (25), malaria (Plasmodium falciparum [*n* = 1], Plasmodium vivax [*n* = 2]) (26), and undetermined fevers (*n* = 9; defined as blood culture negative [23]); PCR dengue-, leptospirosis-, malaria-, and scrub typhus-negative patients were also included. Febrile controls, positive by LAMP but previously negative by R. typhi qPCR, were further evaluated by IFA, as described previously (27). These samples were from patients presenting at Mahosot Hospital with suspected typhus (*n* = 67) and Salavan Provincial Hospital with fever (*n* = 10) (6).

### Prospective evaluation study.

Consecutive inpatients (>15 years old) with clinically suspected murine typhus or scrub typhus presenting to Mahosot Hospital were prospectively enrolled for evaluation of the murine typhus LAMP assay (from May to December 2012; S. Dittrich, unpublished) (*n* = 266). Additional diagnostics included an anti-R. typhi IgM immunoblot assay (ImmunoDOT; GenBio, San Diego, CA, USA), the anti-Orientia tsutsugamushi IgM/IgG rapid test (Standard Diagnostic, South Korea), conventional blood cultures (23), and molecular assays for R. typhi, O. tsutsugamushi (10), and leptospira (25).

### Pathogen isolates.

To evaluate the method, we used DNA extracts from a diverse range of pathogens (*n* = 41), including clinical R. typhi isolates (*n* = 12) and O. tsutsugamushi isolates (*n* = 12) cultured from febrile Lao patients, together with diverse bacterial isolates (*n* = 17) (Burkholderia pseudomallei, Ehrlichia chaffeensis, Escherichia coli, Klebsiella pneumoniae, Leptospira interrogans, Neorickettsia sennetsu, O. tsutsugamushi, P. falciparum, P. vivax, Pseudomonas aeruginosa, Rickettsia conorii, Rickettsia felis, Rickettsia honei, R. prowazekii, Salmonella enterica serotype Enteritidis, S. enterica serotype Typhi, and Staphylococcus aureus) (see Table S1 in the supplemental material).

### Clinical R. typhi isolates.

Rickettsial culture was attempted for patients with a positive anti-R. typhi IgM immunoblot (GenBio, USA) or a positive anti-O. tsutsugamushi IgM/IgG rapid test (Standard Diagnostic, South Korea) of admission sera. Buffy coat samples were cultured with Vero and L929 cell monolayers at 35°C in 5% CO_2_ as previously described (28). DNA extraction was performed using the High Pure PCR template preparation kit (Roche Diagnostics, France) according to the manufacturer's instructions.

### Patient EDTA buffy coat specimens.

DNA was extracted with a QIAamp DNA blood minikit (Qiagen, Germany) according to the manufacturer's instructions, using 200 μl or 100 μl of the buffy coat, with an extended lysis step of 56°C for 1 h and a final elution volume of 100 μl or 50 μl, respectively. The extracted DNA was stored in Tris-EDTA (TE) buffer at −80°C for long-term storage and at 4°C between LAMP/qPCR runs.

### Quantitative real-time PCR assay.

As previously described, a qPCR assay targeting the *ompB* gene (9) was used. Serial dilutions of plasmids (pGEM-T kit; Promega, United Kingdom) used as external controls served for quantification (duplicates), and plasmid copy numbers were calculated using the Quant-iT PicoGreen kit (Invitrogen, USA) according to the manufacturer's instructions.

Bacterial loads were estimated with the following formula: number of R. typhi DNA copies per ml of blood = [(number of copies per PCR mixture using 1 μl DNA template)/2] × 100. Numbers of copies per reaction were calculated using serial plasmid dilutions as external standards (10^3^ to 10^0^ copies/μl), resulting in numbers of copies per μl DNA-eluate (Rotor-Gene 6000 software; Qiagen). The factor 2 adjusts for the 2:1 ratio of blood to DNA-eluate (resulting in numbers of copies per μl buffy coat); the factor 100 corrects for the buffy coat fraction, which makes up ∼10% of the total blood sample (5 ml total collected blood sample, ∼500 μl collected buffy coat fraction [29]).

### LAMP reaction conditions.

All reactions in the development stage of the study were performed in duplicate and singly for the prospective study. The Loopamp DNA amplification kit (Eiken Chemical Co., Ltd., Tokyo, Japan) was used; briefly a 25-μl reaction mixture contained 40 pmol of the FIP and BIP primers, 20 pmol of the Loop-F and Loop-B primers, 10 pmol of the F3 and B3 primers, 12.5 μl of reaction mixture, 1 μl of *Bst* polymerase, 3 μl of template DNA, and distilled water. The reaction mix was incubated in a real-time turbidimeter (model LA-320CE; Eiken Chemical Co., Ltd., Tokyo, Japan) at 60°C for 90 min, followed by enzyme inactivation (80°C for 5 min). Positive and nontemplate controls were included in each run and reliably produced the correct results. Real-time measurement was used, and the first time point at which the change in turbidity increased by 0.1 optical density (OD) unit/s was defined as the positivity criterion.

### Analytical sensitivity and limit of detection of the LAMP assay.

The analytical sensitivity refers to the minimum number of copies in a sample that can be measured accurately with a given assay, also called the limit of detection (LOD). The *C*_95_ endpoint is defined as the concentration of the analyte at which 95% of the evaluated samples test positive (30). Serial 1:5 dilutions of the clinical R. typhi isolates (*n* = 12) were quantitated in duplicate to determine the *C*_95_ LOD. The previously described qPCR assay targeting the single-copy *ompB* gene (9) was chosen due to its reproducibly low LOD (<10 copies/μl DNA template) and its wide use as a validated diagnostic test (6, 31), making it a suitable reference comparator to validate a related test.

### Data analyses.

The sensitivity and specificity of the LAMP assay were determined after assaying the same sample by LAMP and qPCR for two gene targets, the 17-kDa gene (10) and *ompB* (9). Quantitative real-time PCR was used as the gold standard to assess the LAMP assay with a comparable, validated reference test (32). Graphical representations and statistical calculations were performed using GraphPad Prism (version 6 for Macintosh computer, 2012) and/or Stata/SE 10.0 (StataCorp, College Station, Texas).

## RESULTS

### Demographic characteristics.

Samples from 89 patients were included in the development phase; 68.8% were male, and the median age was 38 years (interquartile range [IQR], 23 to 48 years), with a median of 7 days of fever (IQR, 6 to 9 days) prior to admission. During the prospective evaluation, 266 patients were included, of which 56.4% were male, with a median age of 33 years (IQR, 23 to 48 years) and a median of 7 days (IQR, 5 to 10 days) of fever prior to admission. Ages, genders, and numbers of fever days at admission did not differ significantly between the developmental and prospective patient groups.

### Analytical sensitivity (LOD) of the LAMP assay.

Using the isolate dilutions, the *C*_95_ endpoint of the LOD was estimated to be approximately 40 bacterial DNA copies per reaction, as 94.7% of all isolate dilutions above this threshold tested positive. LAMP amplification was, however, obtained for isolates with a number of bacterial DNA copies/reaction less than 10 (median, 80 copies; range, 3 to 420 copies).

### LAMP assay diagnostic sensitivity and bacterial loads in the development phase.

In murine typhus qPCR-confirmed buffy coat samples (*n* = 42), the LAMP assay correctly identified 20/42 samples as containing R. typhi DNA (diagnostic sensitivity, 48%; 95% confidence interval [95% CI], 32.5 to 62.7%). In qPCR- and LAMP-positive (qPCR+/LAMP+) buffy coat samples, the median DNA copy number per ml blood was ∼300 (IQR, 200 to 540 copies). In qPCR+/LAMP− samples, the median copy number per ml blood was ∼180 (IQR, 100 to 330 copies). The difference in bacterial loads between LAMP-positive and -negative samples was statistically significant (*P* = 0.01, Kruskal-Wallis).

According to the Minimum Information for Publication of Quantitative Real-Time PCR Experiments (MIQE) guidelines, qPCR-based detection of samples with fewer than three copies per reaction is unreliable (33). Thus, in a subanalysis, low-positivity samples with <3 copies/reaction (*n* = 8) were excluded from the reference comparator qPCR+ group. Upon exclusion of these samples, the diagnostic sensitivity improved to 53% (95% CI, 36 to 70%), as 5/8 (62.5%) of the low-copy-number samples were LAMP negative.

### LAMP assay diagnostic sensitivity and bacterial loads in the prospective evaluation study.

The prospective evaluation included 266 consecutive patients (Table 2), of which 15 (5.6%) were qPCR positive for R. typhi (9). LAMP assays were positive in 5/15 qPCR-positive samples, resulting in a diagnostic sensitivity of 33% (CI 95%, 9.2 to 56.8%). In qPCR+/LAMP+ samples, the median copy number per ml blood was ∼3,030 (IQR, 410 to 7,250 copies), and in qPCR+/LAMP− samples, the median copy number per ml blood was ∼130 (IQR, 100 to 1,500 copies). The difference in bacterial loads between LAMP-positive and -negative samples was statistically significant (*P* = 0.03, Kruskal-Wallis). No significant difference in clinical presentations was seen between the qPCR+/LAMP+ and qPCR+/LAMP− patients (Table 2).

**TABLE 2:** Clinical features of all patients presenting to Mahosot Hospital as part of the prospective study, analyzed by LAMP positivity^*a*^

Variable	Values for^*b*^:			*P* value^*c*^
	All patients (*n* = 266)	R. typhi PCR-positive patients who were		
		LAMP positive (*n* = 5)	LAMP negative (*n* = 10)	
Median age (yr) (IQR)	33 (23–48)	38 (37–50)	33.5 (26–43)	0.85
No. of males/total (%)	150/266 (56.4)	1/5 (20)	3/10 (30)	1
Median no. of days of fever (IQR) (no. of patients)	7 (5–10) (*n* = 217)	7 (7–8)	7 (6–7) (*n* = 9)	0.10
No. with indicated symptom or sign/total (%)				
Headache	189/220 (85.9)	3/5 (60)	8/10 (80)	0.56
Vomiting	77/218 (35.3)	2/5 (40)	4/9 (44.4)	1
Diarrhea	37/217 (17.1)	0/5 (0)	1/9 (11.1)	1
Rash	18/211 (8.5)	1/5 (20)	2/9 (22.2)	1
Convulsions	19/216 (8.8)	0/5 (0)	0/9 (0)	1
Jaundice	24/216 (11.1)	1/5 (20)	1/9 (11.1)	1
Bleeding	4/76 (5.3)	0/3 (0)	0/5 (0)	1
Myalgia	165/220 (75)	3/5 (60)	8/10 (80)	0.56
Lymphadenopathy	25/213 (11.7)	0/4 (0)	1/9 (11.1)	1
Meningism	24/216 (11.1)	1/5 (20)	1/9 (11.1)	1
Median temp (°C) (IQR) (no. of patients)	38.0 (37.5–38.8) (*n* = 208)	38.4 (37.3–39.3) (*n* = 4)	38.0 (37.6–38.5) (*n* = 9)	0.67
Median Glasgow coma score (range) (no. of patients)	15 (4–15) (*n* = 186)	15 (15–15)	15 (15–15) (*n* = 7)	1
Median pulse/min (IQR) (no. of patients)	94 (82–100) (*n* = 207)	100 (97–104)	96 (91–102) (*n* = 8)	0.61
No. who died/total (%)	5/90 (5.6)	0/3 (0)	0/6 (0)	1
No. who took an antibiotic within the prior week/total (%)	48/116 (41.4)	3/4 (75)	0/4 (0)	0.14
No. of patients positive by the anti-R. typhi IgM immunoblot assay/total (%)	35/200 (17.5)	3/5 (60)	5/8 (62.5)	0.66
Estimated median no. of DNA copies/ml blood (IQR) (no. of samples)	NA	∼3,030 (410–720) (*n* = 4)	∼130 (100–500)	0.03*

a LAMP-positive and LAMP-negative variables were analyzed for differences by the Kruskall-Wallis or Fisher-exact test.

b The available sample size is given in parentheses where the entire sample was not available for a given continuous variable. NA, not available.

c *, *P* < 0.05 (considered significantly different).

After exclusion of 5 out of 14 quantifiable samples due to low copy numbers (<3 copies/reaction) in the reference test, the diagnostic sensitivity improved to 44% (95% CI, 11.9% to 76.9%), as all (5/5) low-copy-number samples were LAMP negative. Thirteen samples from the developmental and prospective data sets were excluded due to very low copy numbers, reducing the total number of available qPCR+ samples to 43. This led to an improvement of the diagnostic sensitivity from 43% (95% CI, 29.9% to 55.9%) to 51% (95% CI, 36.3% to 66.1%).

### R. typhi bacterial loads in patient samples.

**FIG 1:**
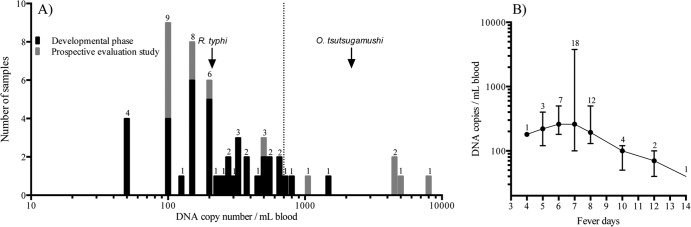
Bacterial loads in patients with acute murine typhus in the Lao People's Democratic Republic (data from quantitative real-time PCR). (A) Histogram depicting the bacterial loads in individual patient samples (*n* = 57) from the development phase (black) and prospective evaluation study (gray). Arrows indicate median bacterial loads for R. typhi (this study) and O. tsutsugamushi (37, 40–42) from Thailand and Lao (S. Dittrich, unpublished); the dashed line represents the approximate 95% LOD of the LAMP assay. (B) Relationship between median (IQR) bacterial loads and numbers of days of fever prior to hospital admission of all available patients with confirmed murine typhus (*n* = 52). The numbers of patients presenting on the different days are indicated above the error bars.

The R. typhi bacterial loads in patient samples with confirmed (qPCR+) acute murine typhus was low, with ∼180 (IQR, 100 to 4,500) and ∼230 (IQR, 150 to 560) copies per ml of blood in the prospective-evaluation (*n* = 14/15) and developmental (*n* = 42/42) phases, respectively. This corresponds to a combined median of ∼210 (IQR, 130 to 500) copies per ml blood in all investigated samples (Fig. 1A).

The highest bacterial loads were found in patients with 6 and 7 days of fever at admission (*n* = 7 and 19, respectively), with bacterial loads of ∼260 (median; IQR, 180 to 500) copies for those admitted with 6 days of fever and ∼260 (median; IQR, 100 to 3,780) copies for those with 7 days of fever. Days 6 to 7 represented the rickettsemic peak, which was followed by a steady decline of R. typhi DNA copy numbers with increasing numbers of days after admission (Fig. 1B).

According to the MIQE guidelines for qPCR experiments, three DNA copies per qPCR is theoretically the lowest detection limit possible (33), and among all R. typhi qPCR+ samples, 13/56 (23.2%) were estimated to have fewer than three DNA copies per qPCR.

### LAMP assay specificity.

With a 90-min assay time, none of the control samples from patients without evidence of R. typhi produced a false-positive result, and the evaluated assay showed an analytical specificity of 100%. Extension of the assay time to 120 min led to a drop of specificity to 92% (95% CI, 85.9% to 98.1%), due to three positive patients and two bacterial isolates (R. conorii, B. pseudomallei) giving positive LAMP results. Two of the positive patients showed serological evidence for a recent murine typhus infection, one with an admission IgM titer of >1:400 and the other with a 4-fold titer rise after 28 days. The third patient was IFA positive for scrub typhus, with an admission IgM titer of >1:400. In the prospective hospital study, the diagnostic specificity of the LAMP assay (90 min) was 98.5% (95% CE, 97.0% to 100%), compared to qPCR diagnostic results. Only 1/5 (20%) of the qPCR−/LAMP+ patients had an alternative diagnosis (S. aureus bacteremia), and all were negative by leptospirosis and scrub typhus qPCR (10, 25). Whether any of these samples were truly false positive could not be determined further, although they were negative in the R. typhi-specific qPCR.

## DISCUSSION

The aim of this study was to establish a simple, inexpensive, sensitive, and specific diagnostic assay for the early diagnosis of murine typhus. LAMP assays are reported to exhibit diagnostic accuracies comparable to those of conventional PCR assays, with the advantage of easier handling (13). In some instances, LAMP has been described to exhibit higher analytical sensitivities than other molecular detection assays (34–36). We hypothesized that a LAMP assay would improve early diagnosis of febrile patients in resource-poor settings, due to rapid, simple, and sensitive detection of R. typhi. However, this first investigation of patient R. typhi bacterial loads suggests that they are too low for the LAMP to be sufficiently sensitive.

The LOD at which 95% of samples could be detected (*C*_95_) was ∼40 DNA copies per LAMP reaction (∼14 DNA copies per μl DNA extract), with reliable ongoing detection of positivity until a cutoff of approximately 10 bacterial DNA copies per reaction was reached, based on results with a dilution series of DNA from cultured R. typhi. This finding is comparable to those from qPCRs and previous LAMP assays for related pathogens, e.g., the LOD for the O. tsutsugamushi LAMP assay with 14 DNA copies per μl DNA extract (37). With both DNA extraction methods used for the preparation of cell culture and clinical samples, it was possible to detect bacterial copies at numbers below 10 copies/ml. Although a direct comparison between the two methods was not possible, both enabled us to detect similarly low pathogen loads, making it unlikely that DNA extraction methods affected the sensitivity of the assay.

The murine typhus LAMP assay demonstrated a high analytical specificity of 100% (95% CI, 100 to 100%) at the optimized assay time but with a low diagnostic sensitivity of 48% (95% CI, 32.5 to 62.7%), which is clinically unacceptable. The proportion of positive specimens detected by LAMP increased from 48% to 53% (95% CI, 36.1% to 69.7%) when specimens with very low bacterial loads (≤3 copies/qPCR assay) were excluded, according to the MIQE guidelines (33).

Although the theoretically low analytical sensitivity/LOD did not translate to good clinical sensitivity, the improvement of the LAMP sensitivity upon exclusion of samples with very small bacterial loads was suggestive of the underlying intrinsic problem. Overall, the R. typhi bacterial loads quantified in Lao human buffy coat samples corresponded to a median copy number of approximately 210 copies per ml of blood [IQR, 130 to 500 copies]. This corresponds to 21 copies per 100 μl buffy coat fraction (∼4 copies/μl DNA) and to approximately 12 copies per LAMP reaction. Indeed, nearly a quarter of all samples from our study population had bacterial loads corresponding to only 1 or 2 bacterial DNA copies per qPCR (∼1 or 2 bacterial DNA copies/μl DNA).

**FIG 2:**
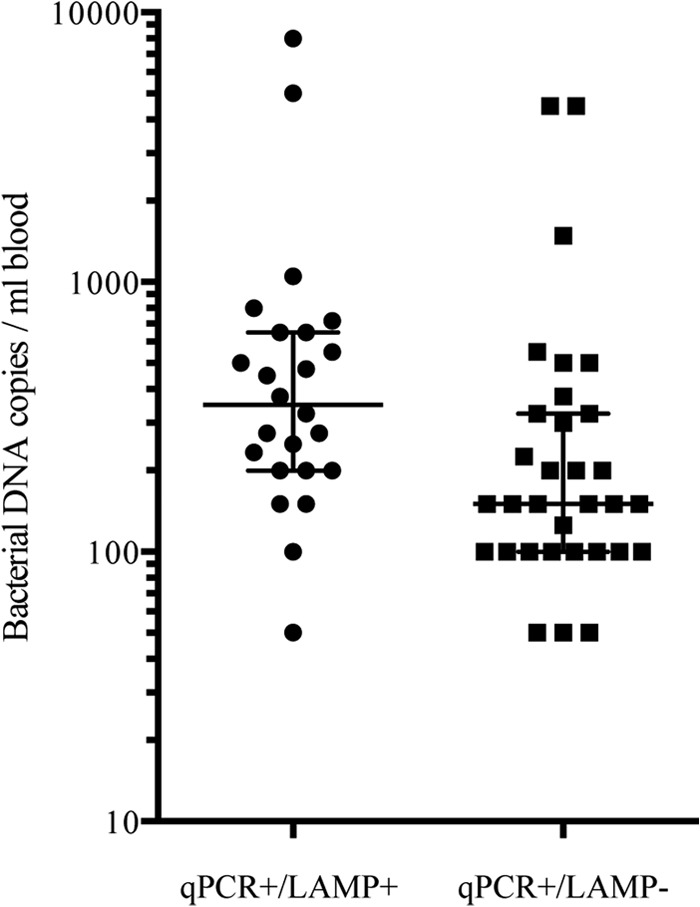
Dot plot depicting the negative effect of low bacterial loads (median, IQR) in patient samples on the R. typhi detection capacity of LAMP. Bacterial load affected the detection of qPCR-positive samples (*n* = 56) by the LAMP assay, as qPCR+/LAMP− (*n* = 32) samples showed a significantly lower bacterial load per ml of blood (median, 150 copies; IQR, 100 to 325 copies) than qPCR+/LAMP+ (*n* = 24) samples (median, 350 copies; IQR, 200 to 650 copies; Kruskal-Wallis, *P* = 0.001).

The low bacterial loads, which even at the peak bacteremic phase commonly remained below the LOD (*C*_95_) of the LAMP assay, appeared to be the reason for the low diagnostic sensitivity of this test. This was further supported by the fact that qPCR+/LAMP− samples were associated with significantly lower bacterial loads than qPCR+/LAMP+ samples in both study arms (Fig. 2), which confirmed that the LAMP assay was working under its LOD. Possible reasons for the suboptimal performance of the LAMP assay may include incomplete separation of PCR inhibitors in the DNA extraction procedure. Although LAMP is widely described as a more robust molecular assay than the gold standard qPCR, it is unlikely that LAMP reactions were inhibited while qPCRs were not (38). It is more probable that high levels of host genomic DNA relative to the low numbers of target DNA negatively affect the reproducibility of positive LAMP results at very low R. typhi DNA copy numbers (39).

These data represent the first report of R. typhi blood loads in humans for this common worldwide infectious disease. We expected to find bacterial loads similar to those in sympatric scrub typhus patients (37, 40–42). O. tsutsugamushi copy numbers in patients in northern Thailand ranged from 1 × 10^6^ to 2.8 × 10^7^ per ml blood (*n* = 7; median, 4 ×10^6^ copies; original reports used numbers of copies/μl blood, which we converted to numbers of copies/ml blood) (41) and in Lao (*n* = 14; median, 2,400 copies) and Thailand (*n* = 7; median, 5,600 copies) ranged from 300 to 2.8 ×10^6^ per ml blood (values for both were originally reported in numbers of copies/μl buffy coat fraction) (37, 40). In a study from northeast Thailand, the median bacterial copy number was 284 per ml blood (*n* = 81; IQR, 124 to 943 copies) (42). Lao patients with scrub typhus who were enrolled at Mahosot Hospital during the same time period as the patients of this study (*n* = 81) presented with a median of 490 copies per ml blood (IQR, 200 to 1,750 copies) (S. Dittrich, unpublished).

Combining all O. tsutsugamushi bacterial load data gives a median of ∼2,200 copies per ml blood. This is ∼10-fold higher than the R. typhi bacterial loads in sympatric patients, albeit from just one murine typhus data set (Fig. 1A).

The broad range of bacterial loads in patients with scrub typhus across studies and geographical regions raises questions as to the effect of bacterial strains, virulence factors, and host susceptibility on admission bacterial loads. It will be interesting to see if a similar breadth of bacterial load ranges is seen in murine typhus patients. Possible explanations for low R. typhi bacterial loads in patients with murine typhus remain to be elucidated but might include low bacterial load inoculation from fleas, rapid postinoculation shift to endothelial and parenchymal compartments, and possible impaired bacterial replication due to antibiotic self-medication prior to hospital admission, although the last was not apparent from our small data set (Table 2). The duration of the rickettsemic phase and number of fever days prior to hospital admission are similar for the two diseases, with approximately 14 days of bacteremia (range, 4 to 125 days) and a median of 7 days of fever (IQR, 7 to 8 days) in murine typhus patients (Fig. 1B) and up to 10 days of bacteremia and 6 days of fever (IQR, 4 to 7 days) in scrub typhus patients (11). This narrow bacteremic window leads to further difficulties in the diagnosis of both rickettsial diseases.

In summary, this study provides evidence that bacterial loads are low in patients with murine typhus, which seems to be unrelated to investigated clinical features (Table 2). This represents a major obstacle for nucleic acid-based diagnostic tests to fulfill WHO's ASSURED criteria for diagnostic tests. Nearly a quarter of samples are within the gray-zone boundaries of <10 copies/PCR, as defined by the MIQE criteria, and as such are by definition not always reliably detectable (33). To overcome the obstacles of low pathogen loads and limited detection windows, alternate gene targets (e.g., multiple-copy gene or interspacer region targets) and novel biomarkers, such as antigens with high blood/serum density, need to be considered. Increasing the sample volume can increase detection capacity but may require an optimization procedure, as the parallel increase in genomic human DNA may negatively affect the assay performance and increasing the sample volume might not be feasible in some clinical settings. An improved understanding of pathogen dissemination dynamics might optimize the timing of specimen collection and the choice of diagnostic strategy, as with other infectious diseases, such as dengue and scrub typhus, where a combination of direct antigen/nucleic acid-based detection with serology has proven useful. In addition, improved and validated sample concentration tools, such as prototypes using disposable microfluidic, dialysis, or affinity chromatography, and innovative detection platforms need to be further developed and subjected to trials in clinical settings (18, 21, 39, 43, 44). Our findings show the importance of pathogen blood density on the final diagnostic accuracy of such assays and underline the importance of combined analytical and diagnostic sensitivity studies.

## Supplementary Material

Supplemental material
