# Supplementary material for: Loop-Mediated Isothermal Amplification for Rickettsia typhi (the Causal Agent of Murine Typhus): Problems with Diagnosis at the Limit of Detection
Source: J Clin Microbiol. 2014 Mar;52(3):832–8. doi: 10.1128/JCM.02786-13 (PMC3957756; doi:10.1128/JCM.02786-13)
Supplement: Supplemental material [file supp_52_3_832__index.html]

Loop-Mediated Isothermal Amplification for Rickettsia typhi (the Causal Agent of Murine Typhus): Problems with Diagnosis at the Limit of Detection — Supplemental material 

# Loop-Mediated Isothermal Amplification for Rickettsia typhi (the Causal Agent of Murine Typhus): Problems with Diagnosis at the Limit of Detection

## Supplemental material

**Files in this Data Supplement:**

- Supplemental file 1 -

  Table S1 (Organisms and relevant strain information for bacterial isolates used during the developmental phase of the study)

  PDF, 107K
