# Supplementary material for: Loop-Mediated Isothermal Amplification for Rickettsia typhi (the Causal Agent of Murine Typhus): Problems with Diagnosis at the Limit of Detection
Source: J Clin Microbiol. 2014 Mar;52(3):832–8. doi: 10.1128/JCM.02786-13 (PMC3957756; doi:10.1128/JCM.02786-13)
Supplement: Supplemental material [file JCM.02786-13_zjm999093197so1.pdf]

Table S1: Organisms and relevant strain information of bacterial isolates used during the developmental phase of the study.

**Organism**	**Strain**	**Organism reference**
Burkholderia pseudomallei	n.a.	Culture isolate[A, B]
Ehrlichia chaffeensis	n.a.	Culture isolate[A]
Escherichia coli	n.a.	ESBL33590[B]
Klebsiella pneumoniae	CDC 298/53	NCTC 9633
Leptospira interrogans	n.a.	Culture isolate[A]
Neorickettsia sennetsu	Miyayama	ATCC VR-367
Orientia tsutsugamushi	Karp	Culture isolate[A]
Orientia tsutsugamushi (n=12)	n.a.	Lao human isolates[A]
Plasmodium falciparum	3D7	Culture isolate[A]
Plasmodium vivax	n.a.	Lao human isolate[A]
Pseudomonas aeruginosa	Boston 41501	ATCC 27853
Rickettsia conorii	Malish	ATCC VR-613
Rickettsia felis	B377 (Australia)	Flea isolate[A]
Rickettsia honei	RB	Human isolate[A]
Rickettsia prowazekii	Breinl	ATCC VR-142
Salomonella enterica serotype Enteritidis	CDC K-1891	ATCC 13076
Salmonella enterica serotype Typhi	Ty 2	NCTC 8385
Staphylococcus aureus	F-182	ATCC 43300

[A]Clinical isolate; identity was confirmed in our or collaborating laboratories using published PCR assays (1-6).
[B]Clinical isolate; identity was confirmed by API NE (bioMérieux; France) and colony morphology.

Supplementary material: References

1. **Henry KM, Jiang J, Rozmajzl PJ, Azad AF, Macaluso KR, Richards AL.** 2007. Development of quantitative real-time PCR assays to detect *Rickettsia typhi* and *Rickettsia felis*, the causative agents of murine typhus and flea-borne spotted fever. Mol Cell Probes **21:**17-23.
2. **Jiang J, Chan TC, Temenak JJ, Dasch GA, Ching WM, Richards AL.** 2004. Development of a quantitative real-time polymerase chain reaction assay specific for *Orientia tsutsugamushi*. Am J Trop Med Hyg **70:**351-356.
3. **Novak RT, Glass MB, Gee JE, Gal D, Mayo MJ, Currie BJ, Wilkins PP.** 2006. Development and evaluation of a real-time PCR assay targeting the type III secretion system of *Burkholderia pseudomallei*. J Clin Microbiol **44:**85-90.
4. **Parola P, Roux V, Camicas JL, Baradji I, Brouqui P, Raoult D.** 2000. Detection of ehrlichiae in African ticks by polymerase chain reaction. Trans R Soc Trop Med Hyg **94:**707-708.
5. **Snounou G, Singh B.** 2002. Nested PCR analysis of Plasmodium parasites. Methods Mol Med **72:**189-203.
6. **Thaipadungpanit J, Chierakul W, Wuthiekanun V, Limmathurotsakul D, Amornchai P, Boonslip S, Smythe LD, Limpaiboon R, Hoffmaster AR, Day NP, Peacock SJ.** 2011. Diagnostic accuracy of real-time PCR assays targeting 16S rRNA and *lipL32* genes for human leptospirosis in Thailand: a case-control study. PLoS One **6:**e16236.
